# Congruence of Microsatellite and Mitochondrial DNA Variation in Acrobat Ants (*Crematogaster* Subgenus *Decacrema*, Formicidae: Myrmicinae) Inhabiting *Macaranga* (Euphorbiaceae) Myrmecophytes

**DOI:** 10.1371/journal.pone.0116602

**Published:** 2015-02-18

**Authors:** Shouhei Ueda, Yusuke Nagano, Yowsuke Kataoka, Takashi Komatsu, Takao Itioka, Usun Shimizu-kaya, Yoko Inui, Takao Itino

**Affiliations:** 1 Department of Biology, Faculty of Science, Shinshu University, 3-1-1 Asahi, Matsumoto, Nagano 390-8621, Japan; 2 Graduate School of Human and Environmental Studies, Kyoto University, Yoshida-nihonmatsu-cho, Sakyo-ku, Kyoto 606-8501, Japan; 3 Graduate School of Global Environmental Studies, Kyoto University, Yoshida-nihonmatsu-cho, Sakyo-ku, Kyoto 606-8501, Japan; 4 Division of Natural Sciences, Department of Arts and Sciences, Faculty of Education, Osaka Kyoiku University 4-698-1 Asahigaoka, Kashiwara, Osaka, 582-8582, Japan; 5 Institute of Mountain Science, Shinshu University, 3-1-1 Asahi, Matsumoto, Nagano 390-8621, Japan; Onderstepoort Veterinary Institute, SOUTH AFRICA

## Abstract

A previously reported mitochondrial DNA (mtDNA) phylogeny of *Crematogaster* (subgenus *Decacrema*) ants inhabiting *Macaranga* myrmecophytes indicated that the partners diversified synchronously and their specific association has been maintained for 20 million years. However, the mtDNA clades did not exactly match morphological species, probably owing to introgressive hybridization among younger species. In this study, we determined the congruence between nuclear simple sequence repeat (SSR, also called microsatellite) genotyping and mtDNA phylogeny to confirm the suitability of the mtDNA phylogeny for inferring the evolutionary history of *Decacrema* ants. Analyses of ant samples from Lambir Hills National park, northeastern Borneo, showed overall congruence between the SSR and mtDNA groupings, indicating that mtDNA markers are useful for delimiting species, at least at the local level. We also found overall high host-plant specificity of the SSR genotypes of *Decacrema* ants, consistent with the specificity based on the mtDNA phylogeny. Further, we detected cryptic genetic assemblages exhibiting high specificity toward particular plant species within a single mtDNA clade. This finding, which may be evidence for rapid ecological and genetic differentiation following a host shift, is a new insight into the previously suggested long-term codiversification of *Decacrema* ants and *Macaranga* plants.

## Introduction

Mitochondrial DNA (mtDNA), owing to its high variability, has become the most popular marker for investigations of the molecular phylogeny, phylogeography, and population genetics of animals during the last three decades. In particular, the mitochondrial gene *cytochrome oxidase I* has been used as an indicator in molecular taxonomy, identification, and DNA barcoding studies [1–3]. However, mtDNA, which is maternally inherited, does not exactly reflect speciation history because of introgressive hybridization among species [4–7]. Therefore, to delimit species boundaries, integration of various types of information, including mtDNA, nuclear DNA (nrDNA), ecological traits, and morphological characters, is necessary [8–10]. It is controversial whether the mtDNA phylogeny of *Crematogaster* (subgenus *Decacrema*) ants, which inhabit *Macaranga* myrmecophytes in Southeast Asia, is congruent with their morphological classification [11–17]. In this study, we examined the congruence between nrDNA simple sequence repeat (SSR, also called microsatellites) genotypes and mtDNA sequences to determine whether the mtDNA phylogeny could be used to infer the evolutionary history of *Decacrema* ants.

**Fig 1:**
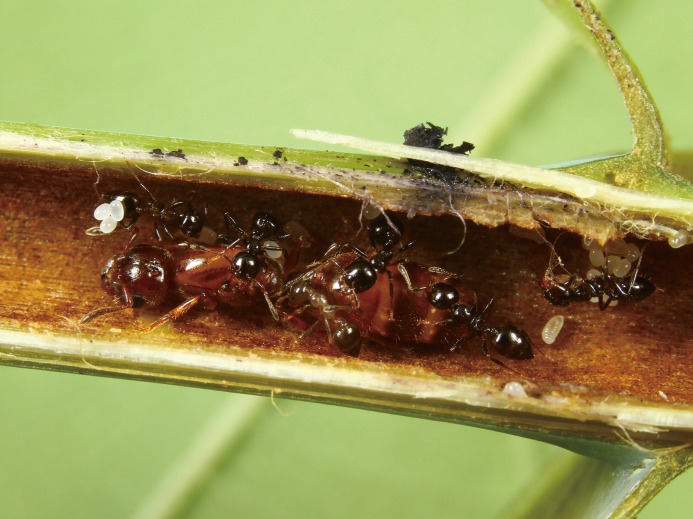
A *Crematogaster* (*Decacrema*) *borneensis* colony nest inside a *Macaranga bancana* stem (photo by T. Komatsu).

About 300 species of genus *Macaranga* are found in the paleotropics from West Africa to the South Pacific Islands [18–20]. Of these, 29 species in western Malesia are myrmecophytes (literally, ant-plants) and provide nesting spaces for symbiotic ants, known as domatia, inside their hollow stems (Fig. 1) [13,21]. In the domatia, a third partner, *Coccus* scale insects, cohabit with the ants [22–25]. The plants also provide food resources, in the form of food bodies secreted by stipules and young leaves and honeydew secreted by the scale insects, for their ants [22,26,27]. The *Coccus* scales settle inside the domatia, where they feed on plant sap and excrete honeydew, which contains sugars and amino acids [22,28]. In return, the ants protect the plants against herbivores and vines [29–32]. Both the ants and the scales are completely dependent upon the host plant and they cannot survive away from it. Therefore, the tripartite interaction is regarded as obligate mutualism [13,31,33,34].

In the early 1990s, it was thought that a single ant species, *Crematogaster borneensis*, might occupy all species of *Macaranga* myrmecophytes, because of a dearth of morphological studies of symbiotic *Decacrema* ants [35]. However, Fiala et al. [13] investigated over 2000 ant queens inhabiting 19 *Macaranga* species throughout the Southeast Asian tropics and provisionally classified the ants into at least nine morphospecies based on queen morphology and life history characters, and they suggested that these ant species usually maintain high host specificity toward *Macaranga* species or species groups.

Itino et al. [14] reconstructed the molecular phylogeny of 47 ants collected from nine *Macaranga* species in northwestern Borneo and the Malay Peninsula by using the nucleotide sequences of mitochondrial *cytochrome oxidase I* (*COI*), and they also, independently of the work of Fiala et al. [13], taxonomically classified these specimens on the basis of worker morphology. Their mtDNA phylogeny included six well-supported clades that were compatible with four morphological species (*C*. *borneensis*, *C*. *decamera*, *C*. sp. 4, *C*. sp. 2). Itino et al. [14] compared the ant phylogeny with the *Macaranga* phylogeny of Davies et al. [21] and found that the association between ants and plants is highly species specific, suggesting possibility of pairwise coevolution between them [14,21].

Feldhaar et al. [11] also conducted a phylogenetic analysis of 34 ant specimens inhabiting 12 *Macaranga* species in northwestern Borneo and the Malay Peninsula based on the nucleotide sequences of mitochondrial *COI* and *COII*, and compared the mtDNA clades with the morphospecies described by Fiala et al. [13]. Their mtDNA phylogeny revealed four distinct clades at a higher taxonomic level that were congruent with morphospecies (msp.) or morphospecies groups (*captiosa* group, *decamera* group, msp. 7 group, and *C*. msp. 8). However, at species level, they detected several mismatches between morphospecies and mtDNA haplotypes.

Quek et al. [16,17] analyzed the *COI* phylogeny of 395 ants inhabiting 22 *Macaranga* species, collected from 32 locations throughout the Southeast Asian tropics spanning Borneo, the Malay Peninsula, and Sumatra. Their mtDNA phylogeny comprised 17 mtDNA clades, most of which could be distinguished from the others by host specificity and distributional range. Estimations of divergence ages based on the phylogeny suggested that the *Macaranga*–*Decacrema* mutualism originated in parallel with the origin of the Southeast Asian tropics (about 20 Mya), and that the partners codiversified synchronously by maintaining their specific association. Quek et al. [16,17] did not examine the concordance between their *COI* phylogeny and morphological groupings, but regarded the mtDNA clades as evolutionarily significant units because each was characterized by unique ecological and distributional traits.

Feldhaar et al. [12] compared morphospecies with their mtDNA phylogeny based on *COI* and *COII*, a haplotype network of nrDNA *elongation factor-1α* (*EF-1α*), and nrDNA SSR genotyping of five microsatellite loci. Their comparison of these four groupings indicated that only the SSR genotyping, not the mtDNA phylogeny or the nrDNA network, delimited morphospecies in *Decacrema* ants. Feldhaar et al. [12] suggested that the maternally inherited mtDNA did not reflect species genealogy owing to ongoing hybridization within the younger *captiosa* group clade, and that the nrDNA haplotype network could not separate the morphospecies because of a lack of mutations in the *EF-1α* gene. Although Feldhaar et al. [12] reported that SSR genotypes were congruent with the delimited *Decacrema* morphospecies, they did not study the concordance between SSR and mtDNA groupings or the host specificity of the ants based on the SSR genotypes.

Here we analyzed SSR and mtDNA sequences of 98 *Decacrema* ants collected from 10 *Macaranga* species in Lambir Hills National Park (LHNP) in northwestern Borneo. The aim of this study was to determine (1) the congruence between SSR and mtDNA groupings and (2) whether ants with similar SSR genotypes significantly preferred a particular host plant group or species.

## Materials and Methods

### Study site and sampling

Sarawak Forest Department provided permission to collect ant samples. Ants were collected from 98 trees representing 10 *Macaranga* species from sections *Pachystemon* and *Pruinosae* at LHNP, Sarawak, Malaysia (4°2'N, 113°50' E, 150–200 m a.s.l.) from 1999 to 2008. Ten workers from each colony were preserved in 99.5% ethanol and constitute one sample. Voucher specimens were deposited at the Faculty of Science, Shinshu University, Matsumoto, Japan. For the mtDNA phylogenetic analysis, we used two species from GenBank as outgroups: (1) a phytoecious *Decacrema* species from Sulawesi that inhabits stem domatia in *Neonauclea*; and (2) a *Crematogaster* species (*C*. cf sp. SKY10) not in the subgenus *Decacrema* that inhabits *M*. *winkleri*, a myrmecophytic species not closely related to sections *Pachystemon* and *Pruinosae*. The two ant taxa are suitable as outgroups for the *Decacrema* inhabitants of *Macaranga*, because the *Decacrema* group inhabiting *Neonauclea* are the sister group of those inhabiting *Macaranga* (Quek et al. 2004) and because *C*. cf sp. SKY10 inhabiting *M*. *winkleri* belongs to the subgenus *Crematogaster* which is taxonomically distant taxa from *Decacrema* (Quek et al. 2004). Collection locations of the specimens and their GenBank accession numbers are listed in S1 Table.

### SSR genotyping

From each ethanol-preserved ant colony sample, DNA was extracted from the whole body of single individual with a DNeasy Blood & Tissue Kit (Qiagen, Hilden, Germany) following the manufacturer’s protocols. Five microsatellite loci (Ca5, Ca12, Ca15, Ca18, and Ca19) developed by Feldhaar et al. [36] were amplified in 98 samples. A multiplex polymerase chain reaction (PCR) analysis was performed with a Type-it PCR master Kit (Qiagen, Hilden, Germany). The PCR temperature profile was 95°C for 10 min, then 25 cycles of 95°C for 30 s, 52°C for 30 s, and 72°C for 1 min, and final extension at 72°C for 60 min. The amplified product was run on an ABI 3130 Genetic Analyzer (ABI, Weiterstadt, Germany), sized relative to Genescan LIZ-500, and genotyped with the GeneMapper* version 4.0 program (ABI, Weiterstadt, Germany).

### STRUCTURE clustering

To detect the nuclear genetic structure from SSR genotype data, we performed a Bayesian model-based clustering analysis with STRUCTURE version 2.3.3 software [37,38]. We ran the admixture model of STRUCTURE for 20 iterations with values of *K* (where *K* is the true number of clusters) from 1 to 10. Each run consisted of a Markov Chain Monte Carlo function performed for 100,000 generations after 100,000 burn-in generations. To detect the most likely value of *K* in the data, we estimated the log likelihood of the data, *lnP(D)*, for each value of *K* across all 20 runs of STRUCTURE and examined an ad hoc quantity, *ΔK*, based on the second order rate of change of the likelihood function with respect to *K* [39].

### MtDNA phylogenetic analysis

The mitochondrial *COI* gene was amplified by PCR with Takara ExTaq* (Takara Bio, Shiga, Japan) using the primers CI-13 (5'-ATA ATT TTT TTT ATA GTT ATA CC-3') and CI-14 (5'-GT TTC TTT TTT TCC TCTT TC-3') [14]. The PCR temperature profile was 35 cycles of 94°C for 30 s, 42°C for 30 s, and 72°C for 90 s. After amplification, the PCR product was purified with ExoSap-IT* (USB, Cleveland, Ohio, USA). Cycle sequencing reactions for both strands were performed with a BigDye* Terminator version 1.1 Cycle Sequencing Kit (ABI, Weiterstadt, Germany) on an ABI 3130 Genetic Analyzer.

*COI* sequences were edited and aligned with the SeqScape* version 2.5 program (ABI, Weiterstadt, Germany). Base-frequency homogeneity was tested by a χ2 test in the Kakusan4 program [40]. The χ2 test did not reject the hypothesis of homogeneity of nucleotide frequencies in each pair of taxa (*P* > 0.50). The degree of substitution saturation in the third codon position of the *COI* sequences was assessed by plotting the transition and transversion rates against genetic distance for each data set with the DAMBE version 5 program by the method of Xia and Xie [41] (S1 Fig.). In the saturation plot analysis, we used the simple JC69 substitution model [42] because DAMBE does not support the J2 substitution model, although in the model selections described in the next paragraph, we used the Jobb (J) 2 substitution model [43]. Substitution saturation in the third codon position was not detected (*P* < 0.001). Additionally, we did not find evidence of any mitochondrial pseudogenes, that is, nuclear mitochondrial transfers (numts), in the mitochondrial *COI* sequences, which can lead to an erroneous phylogeny [44]. The *COI* sequences did not have any indels (small insertions or deletions) or stop codons. As a result, we used all codon positions for the phylogenetic analyses.

We selected the best-fit substitution model for each codon position by using Bayesian information criterion 5 (BIC5) in the Kakusan4 software package [40]. As a result, we selected the following models: J2ef + G for the first codon position; J1 + G for the second codon position; and J2 for the third codon position. We performed a maximum likelihood (ML) analysis with TREEFINDER version October 2008 software [43] and the models selected by Kakusan4. Clade support was assessed by 1000 bootstrap replications in TREEFINDER. In addition, Bayesian posterior probability and maximum parsimony (MP) bootstrap support were obtained with MrBayes version 3.1.2 software [45] and PAUP* 4b10 software [46], respectively. The models selected by BIC5 in Kakusan4 were used in the Bayesian analysis: General Time-Reversible (GTR) [47] for the first codon position; Hasegawa-Kishino-Yano (HKY) 85 [48] for the second codon position; and GTR + G for the third codon position. The Bayesian analysis was run for 1,000,000 generations, with sampling every 100 generations. We assessed the log-likelihood of each sampling point against generation time to identify when the Markov chains reached a stationary distribution, and then discarded the initial 2001 trees as burn-in. The parsimony bootstrap support was assessed with 1000 bootstrap replicates by using heuristic searches with tree bisection and reconnection and 100 random addition replicates for each.

### Host preferences

The *Macaranga* myrmecophytes were divided into three groups according to their stem texture and taxonomic group. Section *Pachystemon*, which has naturally hollow stems and includes 21 myrmecophytic species, was divided into waxy *Pachystemon*, which has waxy crystals on its stem surface, and smooth *Pachystemon*, which has smooth stems that do not secrete wax [21,49]. Section *Pruinosae*, the third group, included five myrmecophytic species with waxy plugged stems that are hollowed out by their ants [21,49].

The preference of each ant genotype for a plant group or species was determined by a one-way χ2 test of the extent of departure of the observed proportion of ants of the genotype using a plant species or species group (the frequency with which ants of the genotype used a plant group or species) from the expected proportion (the plant group or species frequency at LHNP), and 5000 Monte Carlo simulations were also performed to assess the reliability of the χ2 test result. The number of samples varied among the plant groups (waxy *Pachystemon*, *n* = 40; smooth *Pachystemon*, *n* = 46; *Pruinosae*, *n* = 12), plant species (from *M*. sp. A, *n* = 1 to *M*. *bancana* and *M*. *beccariana*, *n* = 15 each), and ant genotypes (from MS2, *n* = 6, to MS5, *n* = 29). These biases reflect the natural frequencies of the plant groups and species and the ant genotypes at LHNP, because we carried out random sampling without regard to the distributions of the plants and ants.

## Results

### SSR genotyping analysis

**Fig 2:**
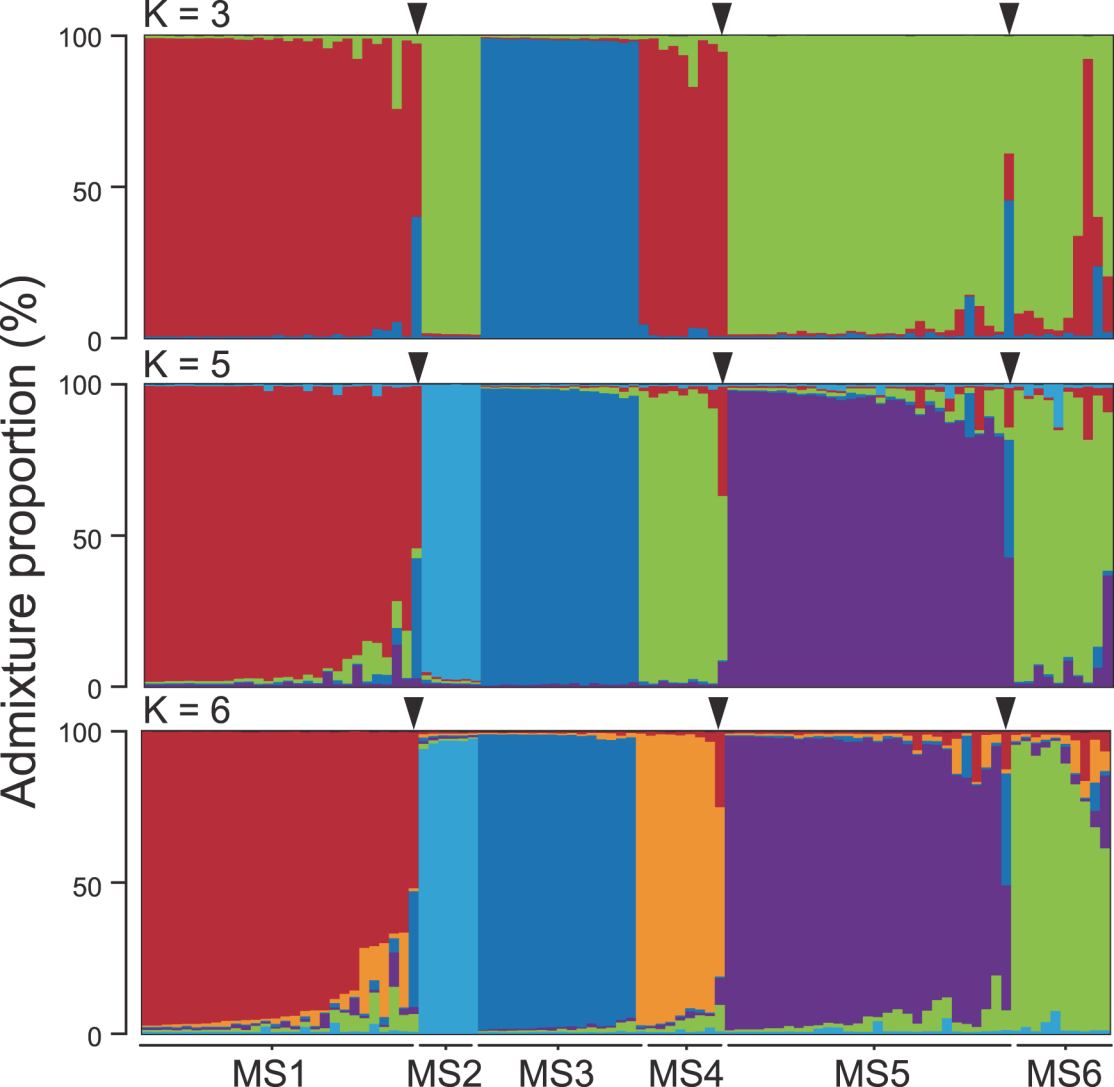
Admixture proportions based on STRUCTURE clustering of five microsatellite loci. Analysis of 98 samples (bars) of *Decacrema* ants inhabiting *Macaranga* in Lambir Hills National Park yielded six genetic clusters (MS1–MS6), which are color coded here for *K* = 3, 5, and 6. Triangles indicate samples that appear to be admixtures of two genetic clusters, suggesting hybridization.

**Fig 3:**
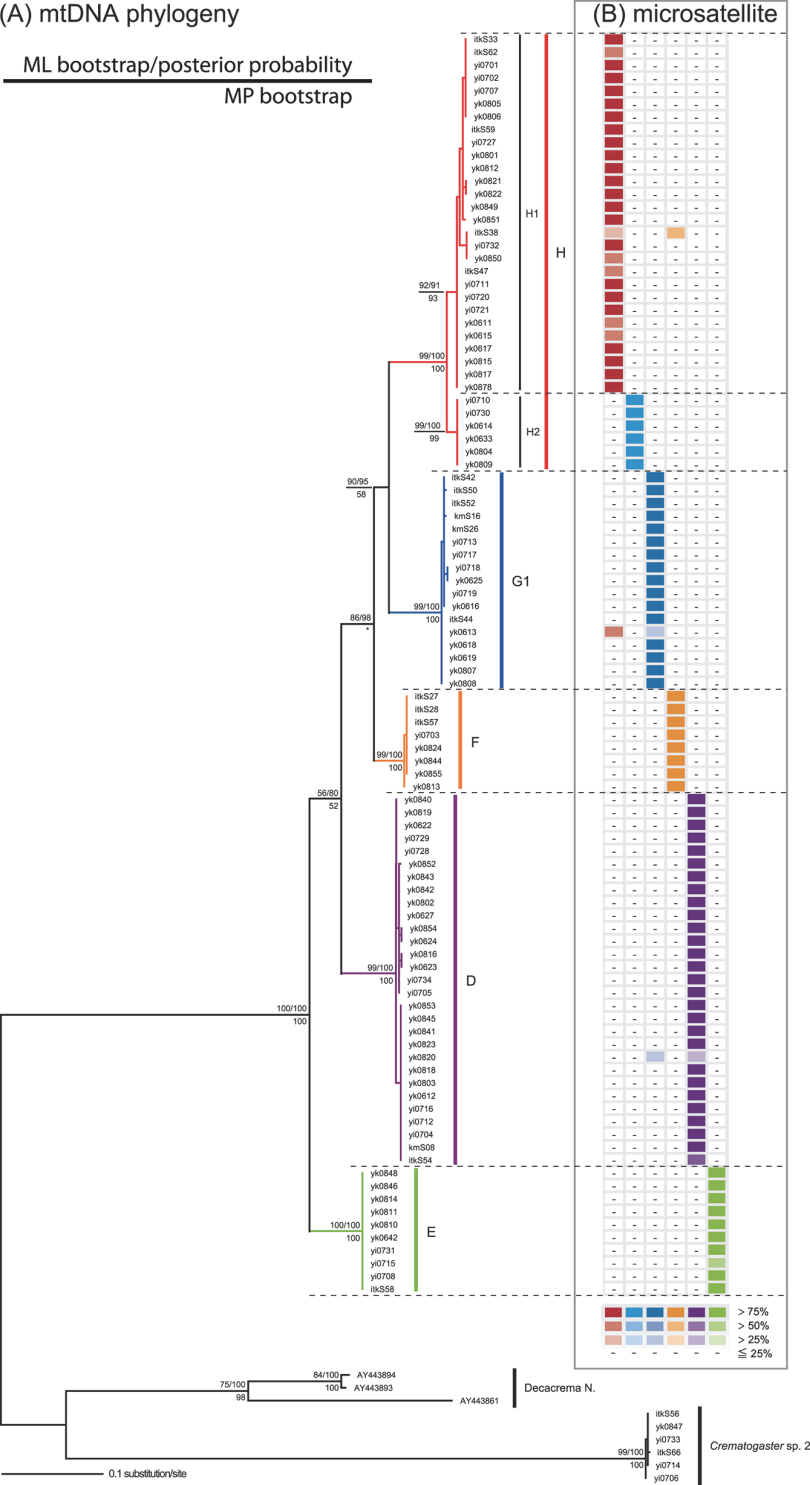
Comparison between the mtDNA phylogeny (A) and nrDNA SSR genotyping (B). The maximum likelihood (ML) phylogeny of 98 samples of *Decacrema* ants inhabiting *Macaranga* in Lambir Hills National Park was inferred from a 569-bp DNA sequence of the mitochondrial gene *cytochrome oxidase I*. The numbers above the branches are the ML bootstrap support/Bayesian posterior probability ratio, and those below the branches indicate the maximum parsimony (MP) bootstrap support. An asterisk (*) replaces one node bootstrap support value because the node was not recovered in the MP bootstrap analysis. The same individuals that were used for the mtDNA phylogeny were genotyped for the five microsatellite loci. The admixture level of each genotype in individual samples is indicated by the color intensity as shown at the bottom of (B).

At least three microsatellite loci were amplified for each ant sample. Non-amplified loci were scored as absent (0) data. STRUCTURE likelihood results were compared with *lnP(D)* and *ΔK*. The highest mean *lnP(D)* was observed for *K* = 5 (*lnP(D)* = -1944.40 ± 26.73 [mean ± SD], *ΔK* = 1.71), whereas the highest *K* was observed for *K* = 3 (*lnP(D)* = -2021.77 ± 2.53 [mean ± SD], *ΔK* = 73.96). In addition to these two estimates of admixture proportions (*K* = 3 and 5), we also conducted the genotyping analysis with the value of K = 6 which is consistent with the number of mtDNA clades (see next paragraph). In spite of relatively low value of mean *lnP(D)* under K = 6 (*lnP(D)* = -1964.65.74 ± 43.66 [mean ± SD]), the SSR genotyping clearly divided the ants into 6 groups (Fig. 2). This low value of *lnP(D)* may be owing to the lack of sampling size of MS4 and MS6 (n = 10 and n = 8, respectively). These three estimates were compatible and the *K* = 6 clustering contained elements of both the clustering of *K* = 3 and *K* = 5 (Fig. 2). Therefore, we decided to adopt *K* = 6 as the most suitable clustering. In addition, some samples appeared to be admixtures of two genetic clusters, whereas each value of *K* was associated with a definite genetic cluster (see Figs. 2 & 3).

### MtDNA phylogenetic analyses

We inferred the molecular phylogeny of the *Decacrema* ants inhabiting *Macaranga* from a 569-bp sequence of mitochondrial *COI* by ML, Bayesian, and MP analyses. The phylogenetic analyses revealed five well-supported primary mtDNA clades that are identical to clades E, D, F, G1, and H of Quek et al. [17] (Fig. 3). Additionally, we identified two highly supported subclades (H1 and H2) within clade H (Fig. 3). Each method yielded a similar topology, but the monophyly of F, G1, and H was not supported by MP bootstrapping, and the monophyly of D, F, G1, and H was poorly supported by ML, MP bootstrapping, and Bayesian posterior probabilities (Fig 3).

### Comparison between SSR genotypes and mtDNA clades

We mapped nrDNA SSR genotypes onto the mtDNA phylogeny of *Decacrema* ants inhabiting *Macaranga* in LHNP (Fig. 3). The degree of congruence between the SSR and mtDNA groupings was 96.9% (95/98). The six groups obtained by STRUCTURE clustering basically corresponded to the six clades obtained by the mtDNA phylogenetic analyses: MS1 corresponded to H1, MS2 to H2, MS3 to G1, MS4 to F, MS5 to D, and MS6 to E (Fig. 3). The three incongruent samples (itkS38, yk0613, and yk0820) exceptionally showed genotypic signals (>25%) derived from two different *Decacrema* clusters (Fig. 3): itkS38 was composed mainly of MS1 (25%) and MS4 (56%); yk0613 was composed mainly of MS1 (52%) and MS3 (38%); and yk0820 was composed mainly of MS3 (37%) and MS5 (41%) (Figs. 2 & 3).

### Specificity

The preference tests of SSR genotypes toward *Macaranga* groups or species indicated that all genotypes showed significant preferences toward or against a particular *Macaranga* group or species (Table 1): MS1 preferred smooth *Pachystemon* and *M*. *trachyphylla* but avoided waxy *Pachystemon*; MS2 preferred waxy *Pachystemon* and *M*. *lamellata* but avoided smooth *Pachystemon*; MS3 preferred *Pruinosae* and *M*. *rufescens* but avoided smooth *Pachystemon*; MS4 preferred smooth *Pachystemon* and *M*. *bancana* but avoided waxy *Pachystemon*; MS5 preferred waxy *Pachystemon* but avoided smooth *Pachystemon* and MS6 preferred smooth *Pachystemon* and *M*. *umbrosa* but avoided waxy *Pachystemon*.

**Table 1:** Results of χ2 tests of the specificity of ant genotypes toward *Macaranga* groups/species.

Ant	n	Plant groups/ species tested	Available	Expected	Observed	P
genotype			hosts	proportion	proportion	
MS1	28	W^a^	W^a^ + S^b^ + P^c^	0.41	0.04	*** (-)
		S^b^	W^a^ + S^b^ + P^c^	0.37	0.96	***
		S^b^	W^a^ + S^b^	0.53	0.96	***
		*M*. *bancana*	W^a^ + S^b^	0.17	0.25	Ns
		*M*. *hullettii*	W^a^ + S^b^	0.14	0.29	Ns
		*M*. *trachyphylla*	W^a^ + S^b^	0.13	0.39	*
		*M*. *umbrosa*	W^a^ + S^b^	0.09	0.04	Ns
		P^c^	W^a^ + S^b^ + P^c^	0.12	0	Ns
MS2	6	W^a^	W^a^ + S^b^ + P^c^	0.41	1	*
		*M*. *lamellata*	W^a^	0.21	1	***
		S^b^	W^a^ + S^b^ + P^c^	0.37	0	* (-)
		P^c^	W^a^ + S^b^ + P^c^	0.12	0	ns
MS3	17	W^a^	W^a^ + S^b^ + P^c^	0.41	0.18	ns
		S	W^a^ + S^b^ + P^c^	0.47	0.12	** (-)
		P^c^	W^a^ + S^b^ + P^c^	0.12	0.71	***
		*M*. *rufescens*	W^a^ + S^b^ + P^c^	0.11	0.65	***
		*M*. sp. A	W^a^ + S^b^ + P^c^	0.01	0.06	ns
MS4	8	W^a^	W^a^ + S^b^ + P^c^	0.41	0	* (-)
		S^b^	W^a^ + S^b^ + P^c^	0.37	1	*
		S^b^	W^a^ + S^b^	0.53	1	*
		*M*. *bancana*	W^a^ + S^b^	0.17	0.75	**
		*M*. *umbrosa*	W^a^ + S^b^	0.09	0.25	ns
		P^c^	W^a^ + S^b^ + P^c^	0.12	0	ns
MS5	29	W^a^	W^a^ + S^b^ + P^c^	0.41	1	***
		*M*. *beccariana*	W^a^	0.38	0.58	ns
		*M*. *hypoleuca*	W^a^	0.25	0.42	ns
		*M*. *havilandii*	W^a^	0.13	0.21	ns
		S^b^	W^a^ + S^b^ + P^c^	0.37	0	*** (-)
		P^c^	W^a^ + S^b^ + P^c^	0.12	0	ns
MS6	10	W^a^	W^a^ + S^b^ + P^c^	0.41	0.1	ns
		W^a^	W^a^ + S^b^	0.56	0.1	** (-)
		S^b^	W^a^ + S^b^ + P^c^	0.37	0.9	*
		S^b^	W^a^ + S^b^	0.53	0.9	*
		*M*. *hullettii*	W^a^ + S^b^	0.14	0.3	ns
		*M*. *umbrosa*	W^a^ + S^b^	0.09	0.5	**
		P^c^	W^a^ + S^b^ + P^c^	0.12	0	ns

The reliability of the tests were assessed by 5000 Monte Carlo simulations.

*P < 0.05

**P < 0.01

***P < 0.001; ns, not significant.

^a^W, waxy *Pachystemon* species (*M*. *beccariana*, *M*. *havilandii*, *M*. *hypoleuca*, *M*. *lamellata*)

^b^S, smooth *Pachystemon* species (*M*. *bancana*, *M*. *hullettii*, *M*. *trachyphylla*, *M*. *umbrosa*)

^c^P, Pruinosae species (*M*. *rufescens*, *M*. sp. A).

## Discussion

### Congruence between SSR genotypes and mtDNA clades

The overall congruence between SSR and mtDNA groupings (Fig. 3) suggests that the mtDNA phylogeny reflects species boundaries and also indicates that the SSR genotyping may certainly delimit the morphospecies of Fiala et al. [13], which were identified on the basis of queen morphology and life history characters [12]. In addition, the genetic resolution of the SSR genotyping and that of the mtDNA phylogeny were almost the same: both methods divided the ants into six groups (Figs. 2 & 3). These results suggest that the *COI* phylogeny is suitable for inferring evolutionary history, phylogeography, and population genetics of *Decacrema* ants inhabiting *Macaranga*, and they are in contrast to the findings of Feldhaar et al. [12], who reported that only SSR genotyping delimited the morphospecies of the ants; the mtDNA phylogeny did not.

Is there a one-to-one correspondence between SSR genotype and *Decacrema* species? Itino et al. [14] documented congruence between the mtDNA phylogeny and species based on worker morphology. We tentatively assigned each SSR genotype to the species identified by Itino et al. [14] based on the species assignment of the mtDNA clades in our phylogeny. On this basis, we assigned MS1, MS2, and MS4 to *C*. *borneensis*; MS3 to *C*. sp. 4; and MS5 and MS6 to *C*. *decamera*. This assignment indicates the absence of a one-to-one correspondence between SSR genotype and species. Thus, it would be desirable to compare the SSR genotypes with the morphospecies of Fiala et al. [13]. At present, however, such a comparison is impossible because the method used to classify the morphospecies has not been published (See [11,12]).

The SSR genotyping is generally used to detect intraspecific genetic variability among individuals or populations with the use of its hypervariable polymorphism. The genotyping conducted here is an exceptional case because it was used to detect genetic variability among species within a subgenus. However, we judged this analysis to be suitable because Feldhaar et al. (2005) designed these SSR markers to delimit the *Decacrema* taxa and STRUCTURE clearly divided the ants into five or six clusterings (Fig. 2). If more *Decacrema* taxa or more samples from other site were added to the analysis, what would happen? If more *Decacrema* taxa from the same site (Lambir) were included, the same result as presented in the manuscript would be obtained: the presented taxa in the manuscript would be divided as it is and the added taxa would form a new grouping. However, if more samples from other sites were added, the same result would not be necessarily obtained. Previous mtDNA phylogenetic analysis revealed that the genetic variation among geographic populations was so high that each ant mtDNA clade was divided into geographic sub-clades (Quek et al. 2007). If more sensitive SSR maker than mtDNA was used, exact clustering may not be reconstructed because geographical genetic variation of SSR may reach saturation and may exceed the variation among species. The SSR genotyping conducted here was successful probably because it excluded geographical variability by using samples collected from a single site. Similar to this, Feldhaar et al. (2010) also conducted the SSR genotyping of the ants by only using samples collected from a limited region, the state of Sabah.

### Hybridization

Although we detected overall congruence between the SSR and mtDNA groupings, we found admixtures of two genetic clusters in three of the 98 samples (Figs. 2 & 3); we attributed these admixture patterns to hybridization between *Decacrema* genotypes. Feldhaar et al. [12,50] also reported hybridization among *Decacrema* ants, about 2% of individuals of the *captiosa* group (this ant group is probably is identical to *C*. *borneensis* of Itino et al. [14]) hybridized. The existence of hybrids can introduce errors into the mtDNA phylogeny, because, as Feldhaar et al. [12] reported, the hybrids may be fertile because hybrid queens can produce workers. However, the high congruence between the SSR genotypes and the mtDNA phylogeny suggests little introgression among genotypes (Figs. 2 & 3). Therefore, hybridization probably may not cause any problematic deviations in the phylogenetic classification based on mtDNA.

To understand how hybridization occurs, it is essential to determine when and where the ants perform their nuptial flights. However, little is known about the environmental cues that lead to nuptial flights in tropical forests [51]. In *Decacrema* ants inhabiting *Macaranga*, it has been inferred that nuptial flights might occur at night because foundress queens were found to colonize saplings at night [35]. But Fiala and Maschwitz [35] reported never seeing a nuptial flight, though they performed numerous checks during both day and night hours. Thus, in a future study, the swarming and mating behaviors of *Decacrema* ants need to be investigated.

### Host preference

Tests of *Decacrema* genotype preferences toward *Macaranga* groups or species in LHNP indicated that all genotypes (MS1, MS2, MS3, MS4, MS5, and MS6) showed significant preferences and avoidances toward particular *Macaranga* groups or species (Table 1). Quek et al. [17] determined host preferences by using about 400 ant samples collected throughout the Asian tropics and identified significant host preferences in the four corresponding mtDNA clades (H, G1, F, D, and E, respectively). Comparison of host preferences test results between this study and that of Quek et al. [17] showed that each genotype and its corresponding mtDNA clade showed a significant preference toward the same plant group or species (e.g., MS1 and H preferred the smooth *Pachystemon*). Several coadaptations between *Decacrema* and *Macaranga* could maintain this high specificity: host selection by foundress queens [52–57]; a trade-off between the host's chemical defense and the biotic defense provided by the ants [29,32,58,59]; and ant adaptations to specialized stem textures of the host [60–62]. The maintenance of high specificity over evolutionary time may promote codiversification in the *Decacrema*-*Macaranga* mutualism [16].

MS1 and MS2 (clades H1 and H2), which together correspond to clade H, showed significant preferences toward different *Macaranga* species: MS1 preferred *M*. *trachyphylla* (smooth *Pachystemon*) and MS2 preferred *M*. *lamellata* (waxy *Pachystemon*) (Table 1). These results suggest that clade H, although identified as a single evolutionarily significant unit, is actually composed of two genotypes, each exhibiting high specificity toward a particular plant species. This result increases the evaluated host specificity between *Macaranga* and *Decacrema*. This finding is evidence for the short-term ecological and genetic differentiation of *Decacrema* ants following a host shift (eg. [63,64]), in contrast to the previously suggested long-term codiversification in the *Decacrema*-*Macaranga* mutualism [65].

## Supporting Information

S1 Fig Relationship between transition (*Ti*, crosses) and transversion (*Tv*, triangles) ratios and genetic distance. The Jukes-Cantor 69 (JC69) distance was used for the substitution model.
(EPS)

S1 Table List of samples. (XLSX)
